# Genomic and phenotypic inconsistencies in *Pseudomonas aeruginosa* resistome among intensive care patients

**DOI:** 10.3389/fcimb.2024.1335096

**Published:** 2024-06-21

**Authors:** Mihails Dolgusevs, Nityanand Jain, Oksana Savicka, Reinis Vangravs, Jevgenijs Bodrenko, Edvins Bergmanis, Dace Zemite, Solvita Selderina, Aigars Reinis, Baiba Rozentale

**Affiliations:** ^1^ Department of Doctoral Studies, Riga Stradinš University, Riga, Latvia; ^2^ Intensive Care Unit, Liepaja Regional Hospital, Liepaja, Latvia; ^3^ Statistics Unit, Riga Stradinš University, Riga, Latvia; ^4^ Joint Microbiology Laboratory, Pauls Stradinš Clinical University Hospital, Riga, Latvia; ^5^ Department of Infectology, Riga Stradinš University, Riga, Latvia; ^6^ Laboratory “Latvian Centre of Infectious Diseases”, National Microbiology Reference Laboratory, Riga, Latvia; ^7^ Faculty of Residency, Riga Stradinš University, Riga, Latvia; ^8^ Central Laboratory, Liepaja, Latvia; ^9^ Department of Biology and Microbiology, Riga Stradinš University, Riga, Latvia; ^10^ Department of Public Health and Epidemiology, Riga Stradinš University, Riga, Latvia; ^11^ Latvian Centre of Infectious Diseases, Riga East Clinical University Hospital, Riga, Latvia

**Keywords:** *Pseudomonas aeruginosa*, environmental reservoirs, intensive care unit, antimicrobial resistance, antimicrobial susceptibility testing, next-generation sequencing

## Abstract

**Objective:**

*Pseudomonas aeruginosa*, a difficult-to-manage nosocomial pathogen, poses a serious threat to clinical outcomes in intensive care (ICU) patients due to its high antimicrobial resistance (AMR). To promote effective management, it is essential to investigate the genomic and phenotypic differences in AMR expression of the isolates.

**Methods:**

A prospective observational study was conducted from July 2022 to April 2023 at Liepaja Regional Hospital in Latvia. The study included all adult patients who were admitted to the ICU and had a documented infection with *P. aeruginosa*, as confirmed by standard laboratory microbiological testing and short-read sequencing. Since ResFinder is the only sequencing-based database offering antibacterial susceptibility testing (AST) data for each antibiotic, we conducted a comparison of the resistance profile with the results of phenotypic testing, evaluating if ResFinder met the US Food and Drug Administration (FDA) requirements for approval as a new AMR diagnostic test. Next, to improve precision, AST data from ResFinder was compared with two other databases – AMRFinderPlus and RGI. Additionally, data was gathered from environmental samples to inform the implementation of appropriate infection control measures in real time.

**Results:**

Our cohort consisted of 33 samples from 29 ICU patients and 34 environmental samples. The presence of *P. aeruginosa* infection was found to be associated with unfavourable clinical outcomes. A third of the patient samples were identified as multi-drug resistant isolates. Apart from resistance against colistin, significant discrepancies were observed when phenotypic data were compared to genotypic data. For example, the aminoglycoside resistance prediction of ResFinder yielded a major errors value of 3.03% for amikacin, which was marginally above the FDA threshold. Among the three positive environmental samples, one sample exhibited multiple AMR genes similar to the patient samples in its cluster.

**Conclusion:**

Our findings underscore the importance of utilizing a combination of diagnostic methods for the identification of resistance mechanisms, clusters, and environmental reservoirs in ICUs.

## 1 Introduction

*Pseudomonas aeruginosa* is a non-fermenting nosocomial pathogen of the ESKAPE group that poses a significant threat in healthcare settings. The pathogen is challenging to treat due to its high levels of antimicrobial resistance (AMR) and is linked to a greater risk of mortality in intensive care units (ICUs) worldwide. In laboratories, antibacterial susceptibility tests (AST) generate phenotypic susceptibility results based on which targeted antimicrobial therapy is prescribed, but this process takes two to three days, depending on the rate of bacterial growth. During this time, patients are prescribed empiric broad-spectrum antibiotics, resulting in temporary overtreating or undertreating of patients along with potential for development of other adverse events.

On the other hand, while genotypic-based point-of-care techniques show potential for rapidly and accurately detecting and confirming AMR, significant advancements are needed to improve their clinical practicality and accessibility. Enriching the existing knowledge base and the tools available for Next-Generation Sequencing (NGS)-based phenotypic AST prediction is essential to address the pressing and rapidly growing clinical needs. For example, reducing the turnaround times for whole genome sequencing and analysis workflows, as well as implementing methodologies that enable direct sequencing on clinical samples, which is particularly important for samples that are normally sterile, such as blood, urine, and cerebrospinal fluid (Deurenberg et al., 2017; Verschuuren et al., 2022).

Furthermore, presently it is difficult to determine which resistance mechanisms are under-expressed or over-expressed, as well as how various combinations of resistance impact the AST results. These questions hold significant importance in clinical practice, as phenotypic-based AST according to the European Committee on Antimicrobial Susceptibility Testing (EUCAST) guidelines offers more nuanced results than simply labelling bacteria as susceptible or resistant to antibiotics. Notably, the guidelines include a category for ‘susceptible with increased exposure’ (I; previously labelled intermediate) (The European Committee on Antimicrobial Susceptibility Testing, 2022; The European Committee on Antimicrobial Susceptibility Testing, 2023). In this context, various efflux pumps, resistance gene regulation pathways, and other mechanisms of resistance play a crucial role in determining the resistance profile of the specimen.

To our knowledge, there is no existing published data that compares phenotypic AST with NGS resistance profiles, particularly analysing discrepancies between these two methods in the Baltic states (Latvia, Lithuania, and Estonia). Consequently, we conducted a pilot investigation on *P. aeruginosa* isolates from ICU patients at a tertiary hospital in Latvia to generate and compare AST and NGS data. We believe the results of this study are especially significant for hospitals and laboratories that provide additional expertise in NGS data analysis. The insights obtained from this research could be crucial in facilitating the adoption of NGS-AST as an auxiliary approach for managing challenging clinical scenarios and enhancing epidemiological surveillance in the forthcoming years.

## 2 Materials and methods

We conducted a prospective observational single-centre study at Liepaja Regional Hospital in Latvia, a tertiary care facility with 349 beds and 10 ICU beds. The hospital handles approximately 15,000 annual hospital admissions and 700 annual ICU admissions. The present study was approved by the Medical and Biomedical Research Ethics Committee of the Riga East University Hospital Support Foundation (N° 8-A/22, 26.07.2022). Patients or their relatives provided signed consent forms for inclusion of the clinical data in the present study.

### 2.1 Study design and inclusion criteria

We performed an epidemiological and molecular analysis of the infection rate and characteristics of *P. aeruginosa* infections in patients admitted to the ICU from July 2022 to the end of April 2023. All adult patients (≥ 18 years) who were admitted to the ICU, irrespective of the diagnosis, and in whom *P. aeruginosa* infection was documented by standard laboratory microbiological testing during ICU admission process or ICU stay were included as per our inclusion criteria. Patients not fulfilling these criteria or with documented *P. aeruginosa* infection before transfer to the ICU were excluded from our analysis.

Multiple samples from the same patient were considered as separate isolates if there was a difference in phenotypic resistance profile against different antibiotic classes. The following clinical data was collected for each patient – age, gender, reason of ICU admission, specimen type and sampling date, duration of ICU stay, duration of hospital stay, and final clinical outcome. We also collected the financial data for hospitalization for these patients from the hospital records. Apart from the clinical samples, we also collected and analysed environmental samples collected on two different occasions – January 2023 (17 samples) and March 2023 (17 samples). These samples were collected from different locations in the ICU including recycle bin lid, preparation room sinks, and patient room sinks.

### 2.2 Sample collection and transport

Clinical samples were collected by the ICU nurses or clinicians and sent for microbiological investigation to the laboratory in accordance with the local hospital protocols. The laboratory is accredited according to the LVS EN ISO 15189:2013 standard. Collected samples included bronchoalveolar lavage, tracheal aspirate, urine, blood, wound material, and surgical site material. Specimens were processed according to the relevant EUCAST guidelines. We excluded samples with improper labelling and those with inadequate patient identifiers. For the environmental samples, surface swabs were collected from the relevant locations in the ICU and processed based on EUCAST guidelines.

### 2.3 Microbiological isolation and identification

The isolation of all *P. aeruginosa* specimens was performed on Columbia Agar with 5% Sheep Blood (BD BBL Becton Dickinson GmbH, Germany). Genus and species identification was done using bacteriological tests based on morphological, culture, and biochemical characteristics like Gram staining and VITEK-2 analyser (BioMerieux, France).

### 2.4 Antibiotic susceptibility testing

Agar disk diffusion method was used for antibiotic susceptibility testing using the Mueller-Hinton II LAB-AGAR™ (Biomaxima, Poland) and the BD BBL Sensi-Disc™ Becton (Dickinson, USA) disks. Susceptibility results were interpreted according to the EUCAST guidelines for *P. aeruginosa.* Susceptibility testing was done against the following antibiotics: AMK – Amikacin; TOB – Tobramycin; CAZ – Ceftazidime; CIP – Ciprofloxacin; IPM – Imipenem; MEM – Meropenem; and TZP – Piperacillin/Tazobactam. Breakpoint tables for interpretation and zone diameters were based on v12.0 (from 01.01.2022 to 31.12.2022) (The European Committee on Antimicrobial Susceptibility Testing, 2022) and v13.0 (from 01.01.2023 to present) (The European Committee on Antimicrobial Susceptibility Testing, 2023). The breakpoint tables are summarized in Supplementary File 1. For *P. aeruginosa*, there were no differences noted in breakpoints and MICs between the two versions.

For colistin (COL), the minimum inhibitory concentrations (MICs) were determined by the VITEK-2 analyser. VITEK-2 analyser (AST-N331) uses broth microdilution method for determining AMR results for colistin. However, according to the manufacturer, this method has no specific indications for use by the U.S. Food and Drug Administration (FDA), and the results obtained for *P. aeruginosa* isolates must be validated and retested using alternative approved techniques. Hence, the frozen cultures were thawed, re-cultivated, and re-tested for colistin resistance using the broth microdilution method (Micronaut MIC-Strips, Bruker, Germany) to enhance the reliability of the results.

### 2.5 DNA extraction and concentration

DNA was extracted from gram-negative *P. aeruginosa* bacterial isolates using the DNeasy Blood & Tissue Kit (QIAGEN, Germany), in accordance with the manufacturer’s protocol. DNA concentration was assessed with a Qubit 4 Fluorometer (Thermo Fisher Scientific, USA) and the Qubit dsDNA BR Assay Kit (Thermo Fisher Scientific, USA).

### 2.6 Library preparation and next-generation sequencing

Libraries were prepared using the Illumina DNA Prep kit (Illumina, USA) according to manufacturer’s protocol. The prepared libraries were quantified with Qubit dsDNA HS Assay Kit (Thermo Fisher Scientific, USA) and size distribution was estimated using TapeStation 4200 with the High Sensitivity D1000 Screen Tape (Agilent, Germany). Libraries were then pooled in equimolar amounts and further quantified by quantitative polymerase chain reaction (qPCR) with the Colibri Library Quantification Kit (Thermo Fisher Scientific, USA). The pooled libraries were then sequenced either on the Illumina NextSeq 550dx (Illumina, USA) or Novaseq 6000 (Illumina, USA) platform in 150PE or 250PE configurations, yielding on average approximately 13 million paired end reads per sample.

### 2.7 Bioinformatics analysis

Bioinformatic analysis was carried out on a high-performance computing cluster using an in-house pipeline – Ardetype v0.0.1 (Bodrenko and Vangravs, 2023). The obtained reads were filtered with Fastp 0.22.0 (Chen et al., 2018) using default parameters, followed by removal of potential human DNA contamination using human reference genome GRCh38.p13 with Kraken2 v2.1.2 (Wood et al., 2019). The filtered reads were then assembled using Shovill v1.1.0 (Seemann et al., 2020) and evaluated with QUAST v5.0.2 (Mikheenko et al., 2018) using the default parameters.

The presence of antimicrobial resistance genes and point mutations were identified with AMRFinderPlus v3.10.42 (database version 2022–10-11.2) (Feldgarden et al., 2021), ResFinder v4.1.11 (database version 2023–03-29) (Bortolaia et al., 2020), and RGI v5.2.1 (database version card_v3.1.4) (Alcock et al., 2023). All three of these databases were used with contigs and default parameters. Conditions for ResFinder were 0.6 identity and 0.9 coverage and for RGI perfect and strict hits only with contigs > 20,000 base pairs. Afterwards results from different databases were aggregated based on gene ontology, thus allowing us to see which genes were found in more than one database.

Additionally, PlasmidFinder (v2023–03-17) (Carattoli et al., 2014) was utilized to detect plasmids. Upon determination of species with Kraken2, sequences underwent further locus-based typing which included 7-gene MLST v2.19.0 using PubMLST typing schemes (Jolley et al., 2018; Seemann et al., 2022), rMLST using PubMLST RESTful API (Jolley et al., 2018) and cgMLST using chewBBACA v3.1.2 (Silva et al., 2018) with cgmlst.org (Ridom, Germany) *P. aeruginosa* scheme (accessed 2023–08-31) (Tönnies et al., 2021; Ridom GmbH Germany, 2023). Isolates were clustered based on their cgMLST profiles with 15 allele distance as the cut-off for cluster analysis. The phylogeny was inferred from cgMLST results and visualized with GrapeTree v1.5.0 (Zhou et al., 2018) using MSTreeV2 minimum spanning tree algorithm.

### 2.8 Statistical analyses

All collected data was managed using MS Excel spreadsheets. Visualizations were prepared using MS Excel, custom python scripts, and R studio v547dcf86 (build 2023–07-07) for Windows 11. Descriptive statistics were used for quantitative variables. Median and range were preferred as measures of central tendency due to largely non-normally distributed variances in the quantitative data.

## 3 Results

### 3.1 Clinical and demographic overview

From 175 patients admitted to our ICU between July 2022 and April 2023, a total of 1090 clinical samples were collected and tested for presence of various infectious agents and their AMR profiles. *P. aeruginosa* infection was confirmed in 33 samples (3.03% of all samples) from 29 patients (16.57% of all patients). One third of the included patients were female (34%) and the median age of the cohort was 70 years (range 23 - 94 years). Bronchoalveolar lavage accounted for 70% of the samples tested, followed by tracheal aspirate (12%). The median length of hospital stay was 29 days (range 4 - 79 days). Infection with *P. aeruginosa* was linked to unfavourable clinical outcome irrespective of the reason for initial admission to the ICU. 69% of the patients died in the ICU with over 20% needing readmission to the ICU. The median duration of ICU stay was 24 days with a range of 1 – 70 days (**Table 1**).

**Table 1:** Overview of the clinical data for 33 clinical isolates obtained from 29 patients admitted to ICU with confirmed *P. aeruginosa* infection.

Sample ID	Sex	Age (Y)	Sample	Hospitalization Days	No. of ICU Days	Reason for initial ICU admission	Readmission to ICU	Clinical Outcome
1113848	F	64	TA	12	12	Gastrointestinal bleeding	No	Exitus letalis
1157409	M	67	BL	73	64	Unspecified sepsis	Yes	Exitus letalis
7573285	M	60	Urine	59	45	Anaphylactic shock	No	Exitus letalis
7697691			BL					
7783213			Urine					
7573761	M	79	TA	79	41	Rupture of abdominal aortic aneurysm	No	Discharged
7626224	M	79	TA	73	70	Encephalitis	Yes	Exitus letalis
7665384	M	53	BL	29	29	Pneumonia	No	Exitus letalis
7769541	M	79	BL	73	70	Encephalitis	Yes	Exitus letalis
7773029	F	58	BL	43	31	Kidney failure	No	Exitus letalis
7779604	F	90	Blood	47	43	Subdural hematoma	No	Exitus letalis
7834649	M	74	BL	63	13	Acute myocardial infarction	No	Discharged
7842025	F	82	SSM	18	2	Appendicitis	No	Exitus letalis
7902735	M	70	Wound	17	13	Urinary bladder perforation	Yes	Exitus letalis
7967075			BL					
8012996	M	72	BL	28	24	Intracerebral hemorrhage	No	Exitus letalis
8041557	M	70	BL	38	31	Urosepsis	No	Exitus letalis
8192469	M	47	TA	46	24	Chronic heart failure	No	Discharged
8277151	M	39	BL	71	33	Burn injury	Yes	Discharged
8330856	M	70	BL	17	11	Polytrauma	Yes	Exitus letalis
8529192	M	63	BL	15	4	Burn injury	No	Discharged
8544379	M	69	BL	45	42	Unspecified sepsis	No	Exitus letalis
8578219	M	59	BL	5	5	Status epilepticus	No	Exitus letalis
8655771	F	73	BL	15	1	Pneumonia	No	Discharged
8663692	M	65	BL	34	29	Pneumonia	No	Exitus letalis
8817169	M	89	BL	7	4	Pneumonia	No	Exitus letalis
8876020	F	75	BL	49	47	Subdural hematoma	No	Exitus letalis
8933298			BL					
8966578	F	23	BL	4	3	Status epilepticus	No	Discharged
8974735	M	94	BL	15	11	Unspecified sepsis	No	Exitus letalis
8988087	F	78	Wound	25	5	Peritonitis	No	Discharged
9013903	F	81	BL	4	4	Acute coronary syndrome	No	Exitus letalis
9023980	F	27	BL	28	15	Polytrauma	No	Discharged

BL, bronchoalveolar lavage; F, female; ICU, intensive care unit; M, male; SSM, surgical site material during abdominal surgery; TA, tracheal aspirate.

### 3.2 Antimicrobial susceptibility testing – phenotypic characteristics

**Figure 1:**
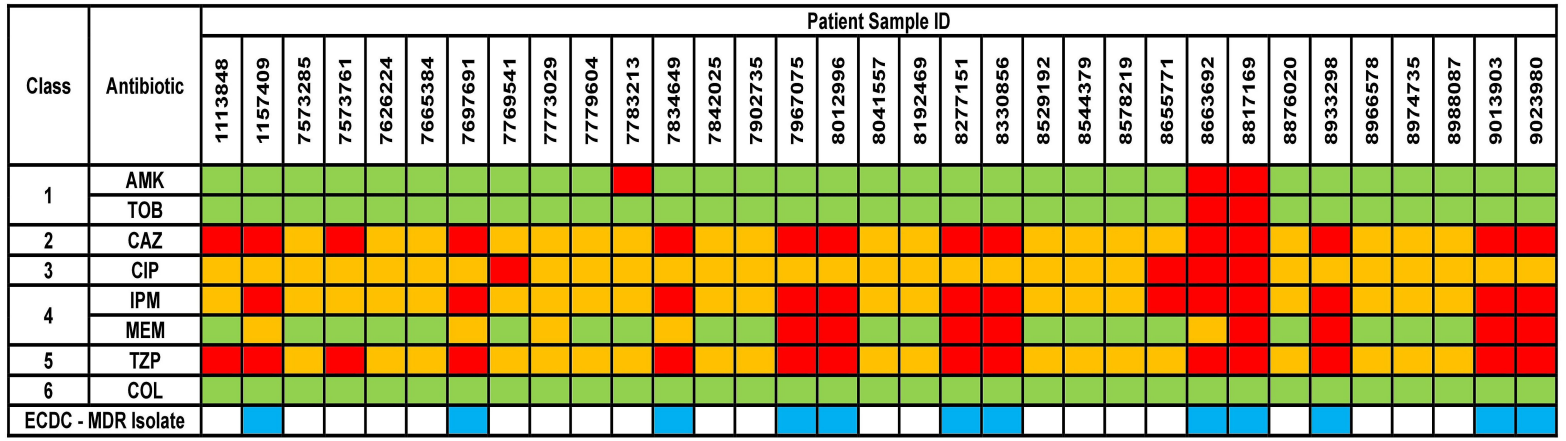
Phenotypical results from the antimicrobial susceptibility testing of the clinical isolates. Green box indicates susceptible isolate, yellow box indicates susceptibility with increased exposure isolate, and red box indicates resistant isolate. AMK, Amikacin; TOB, Tobramycin; CAZ, Ceftazidime; CIP, Ciprofloxacin; IPM, Imipenem; MEM, Meropenem; TZP, Piperacillin/Tazobactam; COL, Colistin. Antibiotic classes are as follows – 1 for aminoglycosides; 2 for cephalosporins; 3 for fluoroquinolones; 4 for carbapenems; 5 for penicillins and 6 for polymyxins. ECDC-MDR isolate (blue box) indicates *Pseudomonas aeruginosa* samples which are resistant to at least 1 antibiotic from a minimum of three or more antibiotic classes – thus known as multi-drug resistant.

The results for AST phenotypic testing for clinical samples are summarized in **Figure 1**. Among the aminoglycosides, 9% and 6% of the samples were resistant to AMK and TOB, respectively. For CAZ and TZP, 42% of the samples were resistant while all other samples showed susceptibility with increased exposure (I). Upon exposure to increased dosage, the number of susceptible isolates increased to 58%. 12% of the samples were resistant to CIP while 39% and 24% were resistant to IPM and MEM, respectively. However, 88%, 61%, and 76% of the isolates were found to show susceptibility upon exposure to increased dosage to CIP, IPM, and MEM, respectively. For COL, VITEK-2 analyser indicated that all samples were susceptible (14 samples had MIC < 0.5 mg/L, 5 samples had MIC 1 mg/L, and 14 samples had MIC 2 mg/K; the cutoff MIC boundary for calling susceptibility was ≤ 4 mg/L). When retested using the broth microdilution method, all samples were indeed found to be susceptible (24 samples had MIC < 1 mg/L while 9 samples that MIC 2 mg/L; the cutoff MIC boundary for calling susceptibility was ≤ 4 mg/L). Accordingly, 36% of the samples fulfilled the criteria for multi-drug resistance (MDR isolates) (Magiorakos et al., 2012).

### 3.3 Plasmid screening

Upon screening for plasmids with Plasmidfinder, only one isolate (ID 8933298) was found to contain a plasmid. This plasmid was identified as similar to pN315 and indicated the presence of blaZ-blaR1-blaI β-lactamase operon, which has been more commonly observed in *Staphylococcus aureus* (Gonzales et al., 2015) isolates. In our case, we believe it might be a remnant of a possible interspecies transmission event.

### 3.4 Characteristics of the constructed NGS resistance profile

The obtained sequencing data demonstrated robust consistency across samples. After quality filtering, the average number of read pairs was 8,148,654 base pairs (bp), with the median of 7,849,376 bp, indicating substantial sequencing depth. The average contig count was 114, compared to a median of 93, demonstrating a thorough genomic assembly. N50 metrics, indicative of assembly quality, yielded an average of 327,695 bp and a median of 308,009 bp, reflecting well-assembled genomic sequences. The average bacterial genome assembly lengths were approximately 6.9 million bases and a median of 6.7 million bases, suggesting uniformity in assembly size. Coverage depth was considerable, with an average of 231× and a median of 221×, ensuring reliable genomic representation. Furthermore, the GC content was consistently recorded at 65.92%, indicating stable nucleotide composition across samples. These metrics underscore the efficacy and precision of the used sequencing and assembly methodologies in accurately capturing the bacterial genomic architecture for our samples.

### 3.5 AST discrepancies with NGS resistance profile

Currently, the most prevalent genomics approach involves identifying known AMR genes from genomic information to predict if a pathogen may be resistant to certain antibiotics. Nevertheless, the catalogue of known AMR genes remains far from comprehensive. *P. aeruginosa*, with its distinct and intricate resistome that is tightly regulated by gene expression, makes NGS-AST a challenging task. In the first step, presence of AMR genes and mutations were assessed using ResFinder (Supplementary File 2). It should be noted that ResFinder is the only database that offers NGS-based AST for each antibiotic, and not just for its class.

For aminoglycosides AMK and TOB, there was 97% and 100% concordance in the resistance profiles, respectively. Regarding CAZ, 19 samples displayed susceptibility with increased exposure (I) in AST but were identified as resistant in NGS data, indicating only 42% concordance. However, the lowest concordance was noted in the case of CAZ, with only 10% of the AST results agreeing with the NGS data. All 13 samples which were marked resistant to IPM in AST were found to be susceptible in NGS data, while for MEM, all 13 samples which were susceptible in AST were resistant in NGS data. Regarding TZP, all 14 resistant AST samples were found to be susceptible in NGS data. A 100% concordance in results was achieved for COL between NGS and phenotypic testing methods.

Clearly, ResFinder exhibited inadequate performance for our *P. aeruginosa* samples, manifesting high rates of very major errors (VME), major errors (ME), and minor errors (mE) which do not meet the US FDA requirements for VMEs < 1.5%, MEs < 3%, and mE ≤ 10%, necessary for the approval of a new AMR diagnostic test or device (**Table 2**). In resistance profiling with phenotypic disk diffusion as the reference baseline, a false-negative outcome or false resistance is deemed a ME while a false-positive outcome or false susceptibility is identified as a VME. A result is classified as minor error (mE) if the disk diffusion shows susceptibility with increased exposure (I) while comparator method shows either resistant or susceptible result.

**Table 2:** Percentage of clinical samples with VMEs, MEs, and mEs based on NGS and AST data (*n* = 33; refer to Supplementary File 2).

AMR Profiling Errors with FDA criteria	Variable	AMK	TOB	CAZ	CIP	IPM	MEM	TZP	COL
Very Major Errors (VMEs) < 1.5%	Number of isolates	0	0	0	0	0	13	0	0
	Percentage	0.00%	0.00%	0.00%	0.00%	0.00%	39.39%	0.00%	0.00%
Major Errors (MEs) < 3%	Number of isolates	1	0	0	2	13	5	14	0
	Percentage	3.03%	0.00%	0.00%	6.06%	39.39%	15.15%	42.42%	0.00%
Minor Errors (mEs) ≤ 10%	Number of isolates	0	0	19	28	0	1	0	0
	Percentage	0.00%	0.00%	57.57%	84.84%	0.00%	3.03%	0.00%	0.00%

### 3.6 Resistance mechanisms and genes

**Figure 2:**
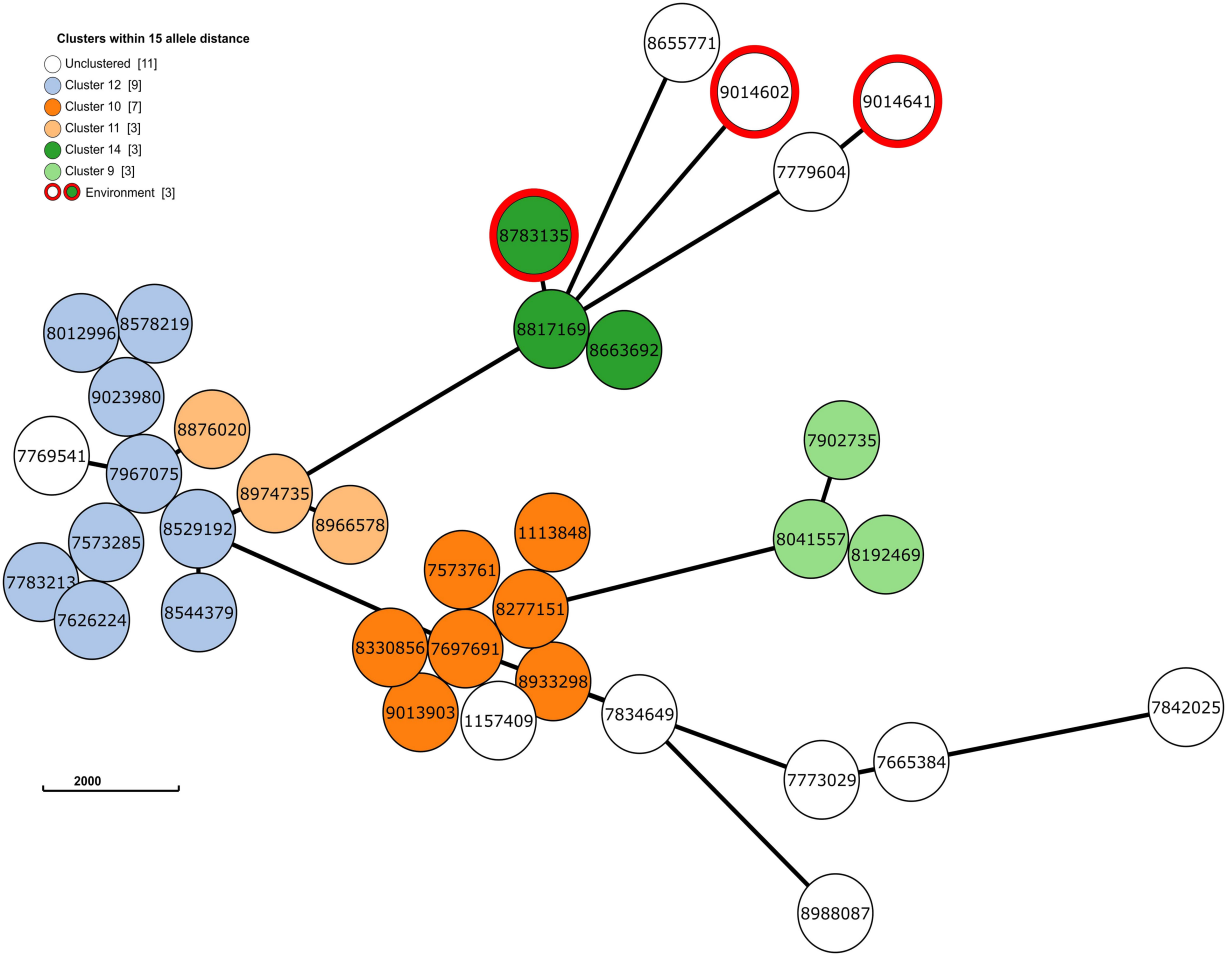
The phylogenetic grape-tree showing the clusters and un-clustered isolates identified from our clinical and environmental samples. Sample ID are shown in the centre of the circles. The tree was prepared using the Grape-tree online render tool (https://achtman-lab.github.io/GrapeTree/MSTree_holder.html).

**Figure 3:**
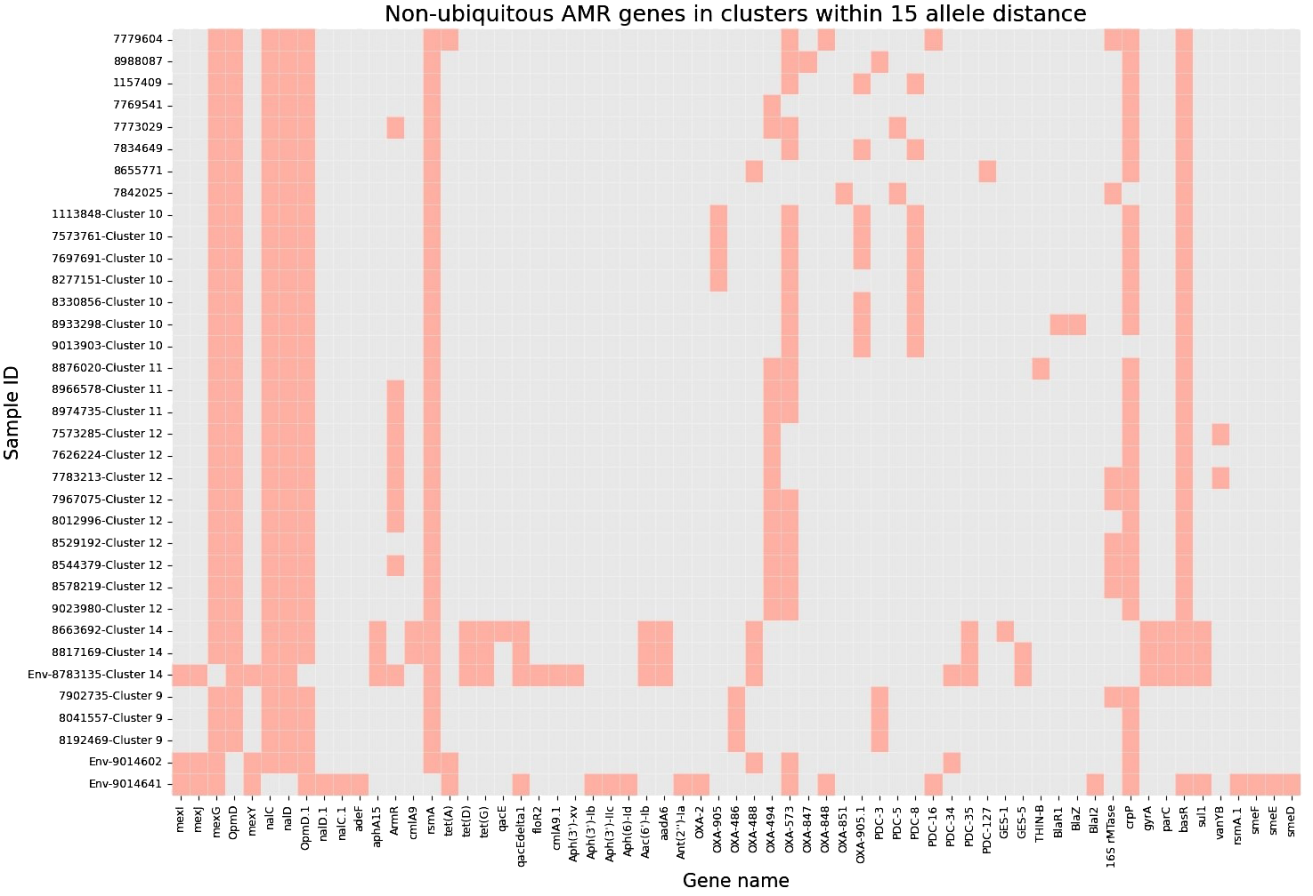
Heatmap demonstrating the presence of non-ubiquitous AMR genes in our 36 isolates (33 clinical and 3 environmental). Samples are grouped based on clusters within 15 allele distance.

Next, to perform NGS-AST with greater precision, we analysed and compared data on AMR determinants from ResFinder with two additional databases, AMRFinderPlus and RGI’s Comprehensive Antibiotic Resistance Database (RGI_CARD), seeking causes for discrepancies between phenotypic resistance profiles. The databases were selected for their broad community adoption – AMRFinderPlus, which is regularly updated by the National Center for Biotechnology Information (NCBI), and RGI_CARD, that is distinguished by its peer-reviewed, high-quality data curation. Each database has its unique strengths which are best complimented by using them in combination. A total of 25 isolates, both clinical and environmental, were grouped into five clusters based on 15 or fewer allele differences among them, with an additional 11 non-clustered isolates (**Figure 2**). **Table 3** summarizes the resistance genes and mechanisms identified in all isolates while **Figure 3** shows the non-ubiquitous AMR genes in our isolates. Supplementary File 3 comprehensively summarizes the list of genes detected by these AMR databases.

**Table 3:** List of antimicrobial resistance genes present in all *P. aeruginosa* isolates based on data from different databases.

Mechanism: Antibiotic Efflux (RGI_CARD)						
*mexA*	*mexG*	*mexP*	*mexW*	*PmpM*	*MuxB*	*CpxR*
*mexB*	*mexH*	*mexQ*	*mexZ*	*OpmB*	*MuxC*	*rsmA*
*mexC*	*mexK*	*mexR*	*oprJ*	*OpmD*	*nalC*	*Type B NfxB*
*mexD*	*mexL*	*mexS*	*oprM*	*OpmE*	*nalD*	TriA*
*mexE*	*mexM*	*mexT*	*oprN*	*OpmH*	*Bcr-1*	TriB*
*mexF*	*mexN*	*mexV*	*emrE(Pae)*	*MuxA*	*YajC*	TriC*
Mechanism: Antibiotic Target Alteration and Antibiotic Efflux (RGI_CARD)						
*cprR*	*cprS*	*soxR*	*basS*			
Mechanism: Antibiotic Efflux and Reduced Permeability to Antibiotics (RGI_CARD)						
*ParR*	*ParS*					
Mechanism: Antibiotic Inactivation (AMRFinderPlus, ResFinder, RGI_CARD)						
*Aph(3’)-IIb*	*catB7*	*FosA*				
Mechanism: Antibiotic Inactivation (ResFinder)						
*PAO*						
Mechanism: Antibiotic Target Alteration (RGI_CARD)						
*arnA*						

* These genes confer the isolates resistance against commonly used surface disinfectants used in the ICUs for disinfection.

#### 3.6.1 Resistance to β-lactams

Although different classes of β-lactamases were detected in the isolates, no strong correlations with phenotypic-based resistance profiles were found – *blaOXA (573, 494, 905), blaPAO, blaPDC, blaGES, blaR1, blaZ, blaTHIN-B*. One sample (ID 8817169) expressed the *blaGES-5* gene which is known to be challenging to treat while also increasing the possibility of nosocomial transmission in hospital environments (Ahmed, 2022).

#### 3.6.2 Resistance to fluoroquinolones

Two isolates (ID 8663692 and 8817169) showed no evidence of AMR during NGS-AST analysis but were found to be phenotypically resistant to fluoroquinolones (Supplementary File 2). Both isolates were found to have *gyrA* and *parC* gene mutations, which are known to cause higher levels of fluoroquinolone resistance (Sionov and Steinberg, 2022; Comprehensive Antibiotic Resistance Database, 2023a). Another isolate (ID 7842025) showed no evidence of AMR during NGS-AST analysis but phenotypically was determined as susceptible, increased exposure (I). This could be attributed to the elevated expression of multiple efflux pumps and the presence of the *CrpP* gene (Peiffer-Smadja et al., 2020; He et al., 2004; Sakhtah et al., 2016; Pang et al., 2019). It is known that minor amino acid variations in antibiotic-modifying enzymes can alter both enzymatic activity levels and substrate specificity. Consequently, the existence of numerous *CrpP* gene alleles could lead to differences in the inactivation of quinolone and the associated activity levels (Ruiz, 2019).

#### 3.6.3 Resistance to aminoglycosides

Two isolates produced the aac(6’)-Ib and aphA15 enzymes, which have clinical significance in many gram-negative pathogens and are associated with higher resistance to aminoglycosides (Pang et al., 2019; Comprehensive Antibiotic Resistance Database, 2023b). One isolate exhibited resistance to AMK but susceptibility to TOB, possibly due to the presence of 16S rRNA methyltransferase and associated methylation, in addition to bacterial pump systems (Costello et al., 2019). The ubiquitous enzyme aph(3’)-IIb (aminoglycoside 3’-phosphotransferases) is not known to confer resistance to AMK and TOB, as it shows poor efficiency in enzyme saturation – TOB is not a substrate of the enzyme, and AMK is a poor substrate (Hainrichson et al., 2007).

#### 3.6.4 Resistance to polymyxins

Regulator protein *pmrA* (*BasR*) was detected in 30 specimens, while *pmrB* (*BasS*) was present in all specimens as a cytoplasmic membrane-bound sensor kinase. Activation and mutation of the *pmrA* gene may result in COL resistance, as previously described in *Klebsiella pneumoniae* and *Salmonella enterica* isolates (Aghapour et al., 2019). It must be acknowledged that our investigation did not extend to the investigation of specific mutations or activation state of genes present in our isolates. Nevertheless, the absence of phenotypic evidence of resistance against COL (**Figure 1**) in our samples led us to conclude that COL resistance may not yet have manifested.

### 3.7 Environmental samples

Apart from the clinical samples, we collected and tested 34 environmental samples at two different time points. Among these samples, only three samples tested positive for *P. aeruginosa* contamination. These samples were gathered from various locations in the ICU – the lid of a recycling bin (sample ID 8783135; Jan 2023), a sink in the medication preparation room (sample ID 9014602; March 2023) and a sink in a patient room (sample ID 9014641; March 2023). For these samples we did not perform phenotypic AST, as we based the analysis within the broader framework of this study, focusing rather on NGS data and identification of clusters involving both patient and environmental samples. Thus, enabling us to identify potential sources of infection in the ICU and improve the infection control and stewardship measures.

**Figure 4:**
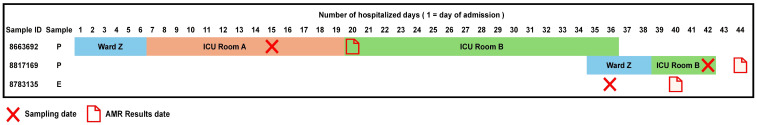
Timeline of events for Cluster 14 samples (2 patient samples and 1 environmental sample).

Accordingly, we found that one of these environmental samples (ID 8783135; lid of the recycling bin) fell in Cluster 14 (**Figure 2**). This sample demonstrated many similarities in terms of the presence of resistance genes with the patient samples (ID 8663692 and 8817169; **Figure 3**) within Cluster 14. For example, the environmental sample also expressed the *blaGES-5* gene and had the *gyrA* and *parC* gene mutations. We then mapped the movement of the patients and this environmental isolate to potentially understand the flow of infection in the ICU (**Figure 4**). It seemed that the recycle bin lid was contaminated with fluids from patient 8663692 and then from the lid, another patient 8817169 acquired the infection. The room was disinfected thoroughly on Day 36 after the death of patient 8663692 and was vacant for the next three days till Day 39 when patient 8817169 was admitted to the room. This made it evident that the bin lid was not sanitized properly.

## 4 Discussion

We herein present the sequencing data using NGS for *P. aeruginosa* clinical and environmental isolates. Predicting which pathogens might exhibit AMR based on genomic data is a promising method for accurately identifying antimicrobial-resistant pathogens. To our knowledge, few papers describe genotypic AST discrepancies in the ESKAPE pathogens group compared to phenotypic AST. Verschuuren et al. (2022), validated NGS-based antimicrobial susceptibility prediction tools for *Escherichia coli* clinical isolates, while Gordon et al. (2014), predicted *Staphylococcus aureus* AMR using NGS. Overall conclusions were similar to ours – NGS-based prediction tools exhibited inadequate performance, showing high rates of mEs, MEs and VMEs, which do not meet the FDA requirements necessary for approving a new AMR diagnostic test. ResFinder’s resistance prediction was almost acceptable for aminoglycosides, meeting the FDA threshold for ME only for AMK at 3.03%. All other antibiotic groups displayed significant discrepancies, except for COL, where all samples were susceptible by both VITEK-2 and broth microdilution. Although a high concordance of COL susceptibility results between VITEK-2 and broth microdilution methods has been observed in previous studies investigating *P. aeruginosa* samples, VITEK-2 has failed to achieve the required FDA criteria for AMR testing (Khurana et al., 2020; Anita, Kumari et al., 2023; Ananda et al., 2024). This necessitates that all samples must be retested with the gold standard i.e., broth microdilution.

Our findings confirm the difficulty of using NGS-AST for *P. aeruginosa*, as mentioned by Mahfouz et al. (2020),. This species poses a significant challenge due to its intricate, extensive, and complex resistome that is closely controlled by various gene expression regulators and efflux mechanisms. The impact of the regulatory effects is still not well understood and may be of critical importance. Kos et al. (2015), revealed that relying solely on NGS-based detection of functional genetic targets proved insufficient in explaining the observed resistance phenotypes in this organism. The development of MDR strains results in a variety of antibiotic resistance mechanisms, rendering standard antibiotics ineffectual for the treatment of *P. aeruginosa* infections. In our opinion, the reality lies somewhere in between subjective evaluations. We still need phenotypic antimicrobial susceptibility testing (AST) as the gold standard, as it is required to guide patient therapy. However, genetic AST can offer valuable additional information in epidemiological outbreaks or complex cases of MDR as an advanced method.

Currently, NGS-based AST is not expected to fully replace phenotypic methods. Genotypic AST shows promise; however, until NGS is fully automated, skilled laboratory personnel will still be needed to prepare, conduct, and interpret genomic results to provide clinicians with reliable information. Although turnaround times are decreasing, it still takes longer than phenotypic AST, particularly when analysis is performed off-site and needs to be sent to a reference centre. Nonetheless, it is a highly promising tool, with potential for comprehensive adaptation for clinical use but it is unlikely it will be implemented in ICU setting in it’s short-read, high throughput, centralized core testing laboratory format. The present study lays the knowledge foundation for implementing long-read based point-of-care sequencing of primary patient samples in conjuncture with host DNA depletion and software-based enrichment methods.

In the case of *P. aeruginosa*, long-read sequencing and the relative ease of library preparation could significantly decrease turnaround times and allow for near same day results, thus justifying the increased costs. Our current approach mainly offers a more precise insight into the potential transmission routes while providing greater resolution when interrogating challenging MDR cases. Finally, monitoring environmental samples in ICU is crucial for identifying potential reservoirs of *P. aeruginosa*. We managed to isolate it from sinks and recycle bin lids. One of isolates belonged to Cluster 14. Identification and elimination of reservoirs in healthcare setting is essential to manage and mitigate risks of bacterium transmission to patients. Based on our finding, we reviewed the Environmental Service (ES) and Nursing Environmental Cleaning Practises (NECP) at our hospital and conducted staff training including repeating infection control mechanisms. Furthermore, existing monitoring and checking regimens were strengthened.

### 4.1 Limitations

Our study findings are subject to certain limitations. The depth of clinical data analysis was insufficient to unequivocally attribute mortality solely to *P. aeruginosa* infections, disregarding the potential impact of other chronic conditions prevalent in our older patient cohort. However, we observed a notable increase in mortality rates comparing patients with *P. aeruginosa* infections (69%) to the broader ICU population (33%). Second, due to resource constraints, we could not perform repeat culture-AST tests on borderline phenotypes (as defined by EUCAST MIC standards), which may have reduced the incidences of minor, major, and very major errors (mEs, MEs and VMEs), in line with findings from Gordon et al. (2014), who reported a 40% resolution in discrepancies upon re-evaluation. A follow-up study addressing this limitation would, hence, be crucial. Third, we did not extend our investigation to specific mutations or activation state of genes present in our isolates, which could have been insightful in providing a completer picture of the resistome landscape. A more comprehensive epidemiological analysis could have enhanced our understanding of the hospital-wide AMR dynamics.

Finally, resistance profiles can vary from one hospital to another, thereby limiting generalizability of our report. Nonetheless, our pilot study represents the first initiative in Latvia where our hospital collected samples and performed sequencing in the National Reference Laboratory to analyse *P. aeruginosa* resistance profiles. This pilot data serves to provide supplementary expertise in NGS data analysis for our colleagues as currently our medical reference laboratory is the only one in Latvia that works with NGS data. Additionally, this initiates a framework for further analysis of resistance profiles in other Latvian hospitals. This data is also crucial for our hospital’s ICU, as it enriches our phenotypic data, providing a more comprehensive view of the actual situation.

## 5 Conclusions

The amalgamation of diagnostic approaches, including phenotypic and genotypic analyses, enables public health officials to recognize resistance factors and clusters, while minimizing errors in interpretation by scrutinizing discrepancies between the two methods. Accurate AST is crucial for improving antimicrobial stewardship and clinical outcomes, even in the face of increasing global resistance. Current and forthcoming NGS studies on bacterial resistome will enhance clinical knowledge and enable more targeted and effective use of antibiotics, despite the high percentage of discrepancies between phenotypic and genotypic AST methods. Nosocomial *P. aeruginosa* infection, especially MDR *P. aeruginosa* is associated with substantial clinical and economic burden. Done in a timely manner, pathogen identification and AST testing (both phenotypic and genotypic) along with the wise use of antibacterial therapy, could enable clinicians to treat such infections promptly, thereby decreasing the financial burden on the healthcare systems.

## Data availability statement

Sequencing data are publicly available at the European Nucleotide Archive (ENA) with project accession number PRJEB73784.

## Ethics statement

The studies involving humans were approved by Medical and Biomedical Research Ethics Committee of the Riga East University Hospital Support Foundation (No 8-A/22, 26.07.2022). The studies were conducted in accordance with the local legislation and institutional requirements. The participants provided their written informed consent to participate in this study.

## Author contributions

MD: Conceptualization, Data curation, Formal analysis, Funding acquisition, Investigation, Methodology, Project administration, Resources, Software, Validation, Writing – original draft, Writing – review & editing. NJ: Conceptualization, Data curation, Formal analysis, Funding acquisition, Validation, Visualization, Writing – original draft, Writing – review & editing. OS: Investigation, Methodology, Validation, Writing – review & editing. RV: Formal analysis, Investigation, Methodology, Software, Validation, Visualization, Writing – review & editing. JB: Investigation, Validation, Writing – review & editing, Formal analysis, Resources. EB: Investigation, Validation, Writing – review & editing. DZ: Investigation, Validation, Writing – review & editing. SS: Investigation, Writing – review & editing, Validation. AR: Conceptualization, Funding acquisition, Investigation, Methodology, Project administration, Resources, Supervision, Writing – original draft, Writing – review & editing. BR: Conceptualization, Formal analysis, Funding acquisition, Investigation, Methodology, Project administration, Resources, Supervision, Validation, Writing – original draft, Writing – review & editing.
