# Supplementary material for: Genomic and phenotypic inconsistencies in Pseudomonas aeruginosa resistome among intensive care patients
Source: Front Cell Infect Microbiol. 2024 Jun 21;14:1335096. doi: 10.3389/fcimb.2024.1335096 (PMC11224958; doi:10.3389/fcimb.2024.1335096)
Supplement: Supplementary file 1 [file DataSheet_1.pdf]

EUCAST breakpoints for antibiotics against *P. aeruginosa* in MIC and Disk Diffusion methods v12.0 (valid from 01.01.2022 to 31.12.2022)

Antibiotic*	MIC breakpoints (mg/L)			Disk Content (µg)	Susceptible (S) [in mm]			Notes
	Susceptible, standard dosing (S)	Susceptible, increased exposure (I)	Resistant (R)		Susceptible, standard dosing (S)	Susceptible, increased exposure (I)	Resistant (R)	
**AMK**	≤ 16	-	> 16	30	≥ 15	-	< 15	
**TOB**	≤ 2	-	> 2	10	≥ 18	-	≥ 18	
**CAZ**	≤ 0.001	> 0.001 – 8	> 8	10	≥ 50	17 – 49	< 17	
**CIP**	≤ 0.001	> 0.001 – 0.5	> 0.5	5	≥ 50	26 – 49	< 26	
**IPM**	≤ 0.001	> 0.001 – 4	> 4	10	≥ 50	20 – 49	< 20	
**MEM**	≤ 2	> 2 – 8	> 8	10	≥ 20	14 – 19	< 14	
**TZP**	≤ 0.001	> 0.001 – 16	> 16	30 – 6	≥ 50	19 – 49	< 18	**18 – 19 is ATU – area of technical uncertainty
**COL**	≤ 4	-	> 4	-	Notes	Notes	Notes	Use an MIC method

EUCAST breakpoints for antibiotics against *P. aeruginosa* in MIC and Disk Diffusion methods v13.0 (valid from 01.01.2023 to Present)

Antibiotic*	MIC breakpoints (mg/L)			Disk Content (µg)	Susceptible (S) [in mm]			Notes
	Susceptible, standard dosing (S)	Susceptible, increased exposure (I)	Resistant (R)		Susceptible, standard dosing (S)	Susceptible, increased exposure (I)	Resistant (R)	
**AMK**	≤ 16	-	> 16	30	≥ 15	-	< 15	
**TOB**	≤ 2	-	> 2	10	≥ 18	-	≥ 18	
**CAZ**	≤ 0.001	> 0.001 – 8	> 8	10	≥ 50	17 – 49	< 17	
**CIP**	≤ 0.001	> 0.001 – 0.5	> 0.5	5	≥ 50	26 – 49	< 26	
**IPM**	≤ 0.001	> 0.001 – 4	> 4	10	≥ 50	20 – 49	< 20	
**MEM**	≤ 2	> 2 – 8	> 8	10	≥ 20	14 – 19	< 14	
**TZP**	≤ 0.001	> 0.001 – 16	> 16	30 – 6	≥ 50	19 – 49	< 18	**18 – 19 is ATU – area of technical uncertainty
**COL**	≤ 4	-	> 4	-	Notes	Notes	Notes	Use an MIC method

***Abbreviations: AMK – Amikacin; TOB – Tobramycin; CAZ – Ceftazidime; CIP – Ciprofloxacin; IPM – Imipenem; MEM – Meropenem; TZP – Piperacillin/Tazobactam; COL – Colistin.**
